# High blood pressure pattern amongst adolescents in Lagos, South West Nigeria

**DOI:** 10.11604/pamj.2023.44.206.38670

**Published:** 2023-04-28

**Authors:** Ifeoma Atoh, Joseph Ezeogu, Chinelo Vivian Okeke, Stella Ijeoma Umeh, Ekanem Ekure, Samuel Ilenre Omokhodion, Fidelis Olisamedua Njokanma

**Affiliations:** 1 Department of Paediatrics, College of Medicine, University of Lagos, Nigeria,; 2 Department of Paediatrics, Federal University Teaching Hospital Owerri, Imo, Nigeria,; 3 Department of Paediatrics, Abia State University Teaching Hospital, Aba, Nigeria,; 4 Department of Paediatrics, College of Medicine, University of Ibadan, Ibadan, Nigeria,; 5 Department of Paediatrics, College of Medicine, Lagos State University, Lagos, Nigeria

**Keywords:** High blood pressure, elevated blood pressure, hypertension, adolescent, secondary school

## Abstract

**Introduction:**

high blood pressure (HBP), once considered rare in adolescents is now a growing health problem. Usually asymptomatic in adolescents, if uncontrolled, can track into adulthood leading to various end-organ complications. In 2017, the American Academy of Pediatrics (AAP) published a new Clinical Practice Guideline (CPG) for screening and management of high blood pressure in children and adolescents to update the 2004 Fourth report. The objective of this study was to determine the prevalence of high blood pressure among adolescents in Mushin Local Government Area (LGA) using the 2017 AAP guidelines.

**Methods:**

a descriptive cross-sectional study, conducted from August 2020 to December 2020. A two-stage sampling technique was used to select 1490 students aged 10 to 19 years, from 14 secondary schools. Socio-demographic information and relevant clinical data were obtained using a structured questionnaire. The anthropometry and blood pressure measurements were taken according to standard protocol (elevated blood pressure is systolic and/or diastolic blood pressure ≥ 90^th^ percentile but ≤ 95^th^ percentile for age, gender and height). Socio-demographic and anthropometric characteristics were described with descriptive statistics. Categorical variables were summarized using frequency and percentages, while numerical variables were summarized using mean and standard deviation. The predictors of hypertension were determined using logistic regression analysis.

**Results:**

study participants were 1490, 49.9% (744) were male and 50.1% (746) females (male: female ratio was 1: 1). Subjects mean age was 14.39 ± 2.79 years. There were 8.9% overweight and 1.7% obese participants. Prevalence of high blood pressure, elevated blood pressure and hypertension were 26.7% (n = 398), 13.8% (n = 205), and 12.9% (n = 193). Middle and late adolescence, when compared to early adolescence, significantly predicted the likelihood of high blood pressure; aOR 1.78, 95%CI: 1.20 - 2.63, p=0.004 and 3.90 (2.69 - 5.67, p=0.001 respectively). Similarly, male sex had increased odds for raised blood pressure when compared to female sex aOR 1.49,95% CI: 1.1 - 2.0, p= 0.009.

**Conclusion:**

the prevalence of high blood pressure, elevated blood pressure and hypertension amongst adolescents was high. Early detection and treatment will forestall development of complications.

## Introduction

High blood pressure is a condition in which the blood vessels have persistently raised pressure, putting them under increased stress [1]. If left uncontrolled, high blood pressure could lead to complications such as left ventricular hypertrophy, myocardial infarction, cerebrovascular accident, and renal insufficiency [1]. High blood pressure is currently classified into three groups namely elevated blood pressure, stage 1 and stage 2 hypertension [2]. The previous pre-hypertension is now called elevated blood pressure, while Stage 1 and Stage 2 hypertension retain their nomenclature, but with different cut-off points. The 2017 guideline also has new normative paediatric blood pressure tables based on blood pressures from normal-weight children [3], whereas the Fourth Report included children and adolescents who were overweight and obese, thus creating a bias since these factors have a strong association with high blood pressure [4]. Risk factors for high blood pressure in adolescents include age, gender, obesity, physical inactivity, family history of hypertension in first degree relatives, socioeconomic status, cigarette smoking and alcohol intake [3]. Other risk factors include birth weight, maturity during birth, heredity, and diet renal abnormalities, coarctation of the aorta, medications, neoplasm, etc. [5]. According to the World Health Organization, about 1.13 billion people globally are living with hypertension, and two-thirds of this number resides in low to middle-income countries [6]. The estimated prevalence of high blood pressure in adolescents in a meta-analysis involving studies from five continents including Africa was reported as 11.2% [7]. In Sub-Saharan Africa, the prevalence ranges from 0.2 to 24.8% [8], and in Nigeria, the prevalence ranges from 0.1% to 17.5% [9].

It is increasingly evident that the origin of essential hypertension in adolescents may be traced to early life [10-12]. The increasing incidence of metabolic syndrome [13] and its association with obesity and hypertension need not be overlooked. In addition, the recognition that blood pressure elevation in childhood is a predictor of high blood pressure in adults, have led to renewed curiosity in investigating blood pressure and its correlates in childhood and adolescence [14,15]. For this reason, routine measurement of blood pressure among children and adolescents is recommended [16]. Evaluation of persons with elevated blood pressure which is a risk for hypertension would help in early identification, which would enable prompt intervention and prevention of complications associated with hypertension. This study, sought to determine the prevalence of high blood pressure among adolescents in Mushin Local Government Area using the 2017 AAP Guideline. It was hoped that the findings of this study will provide accurate information on the current burden of high blood pressure among adolescents within Mushin LGA in Lagos using the 2017 AAP Guideline. This data would be useful material for advocacy and policy formulation on screening for high blood pressure in adolescents in this environment to ensure early diagnosis, treatment, and prevention of end-organ damage.

## Methods

**Study design and setting:** it was a descriptive cross-sectional study, conducted in Mushin Local Government Area of Lagos State South-western part of Nigeria from August 2020 to December 2020. It has one educational district called Educational District VI located in the Oshodi area of Lagos State. This study was conducted in 14 (12 private and 2 public) secondary schools in Mushin Local Government Area, among adolescents aged 10 to 19 years over a 5 months period, August to December 2020.

**Inclusion and exclusion criteria:** students whose parents/ guardians gave consent; and or adolescents who gave assent for the study were consecutively enrolled. Adolescents who had renal conditions such as acute glomerulonephritis, reno-vascular and renal parenchymal diseases; and those who were on antihypertensive medications were excluded.

**Sampling procedure:** subjects were 1355 students, selected using a two-stage sampling technique. The sample size was determined using the formula for prevalence studies [17].

n = *Z2P/Qd2*

Where: n = minimum sample size when study population is > 10,000; z = Standard normal deviation corresponding to 95% confidence interval = 1.96; p = prevalence rate of high blood pressure in adolescents from a previous study done in Lagos, Nigeria i.e. 16.5% (0.17) [18]. q = (1 - p) i.e. 0.83; d = precision level. For this study, this was set at 2% (0.02) Substituting these figures into the formula:

n = 1.962 × 0.17 × 0.830.022 = 1355

The minimum sample size was 1355. An additional 10% (135 pupils) was added for non-response. This brought the total calculated sample size for the study to 1490. Thus 1,490 adolescents were recruited for the study.

**Data collection:** within randomly selected schools, participants were recruited from each class determined by proportional allocation using the school´s register; and stratified along gender lines. Subjects were selected from each stratum by simple random sampling. Socio-demographic information and relevant clinical data were obtained using a structured questionnaire. Questions were asked to exclude presence of symptoms of renal disease and history of hypertension in the participants. The anthropometry and blood pressure measurements were taken according to standard protocol. Body mass index Z score was determined using the WHO chart for children 5-19 years [19]. The weight was measured to the nearest 0.1kg with minimal clothing using a standardized weighing scale (SECA model 756). Height was measured with the participant standing straight on bare feet, with both heels placed together, buttocks, shoulder blades and head without headgear in Frankfurt plane [20] in contact with the measuring rule, and readings recorded to the nearest 0.5cm using a stadiometer (SECA model 213). The blood pressure was measured after the subject must have rested in a seated position for 5 minutes legs uncrossed and flat on the floor; using Accoson sphygmomanometer. The measurement was done as recommended in the Seventh Report of the Joint National Committee on Prevention, Detection, Evaluation, and Treatment of High Blood Pressure (JNC-7) [21] with the subjects sitting quietly and the right arm on a table at the level of the heart.

An appropriately sized cuff was snuggly wrapped on the right upper arm after restricting clothing had been removed. The cuff was then inflated to a pressure of 30 mmHg above the level at which the radial pulse was no longer palpable. The stethoscope was placed over the brachial artery in the cubital fossa and the pressure in the cuff was deflated at 2 mmHg per second. The first audible sound (first Korotkoff sound) was recorded as the systolic blood pressure (SBP). The pressure in the cuff was further lowered until the sounds disappear completely. This was the fifth Korotkoff sound, and the corresponding pressure recorded as the diastolic blood pressure (DBP). The blood pressure was measured three times at an interval of 2 minutes and the mean recorded. The systolic and diastolic recording, as well as the age of the patient, the gender and the height in centimeters (cm), were used to obtain the blood pressure percentile for the 2017 AAP Guideline using the MD CALC [22]. Social class was determined using the socioeconomic indices of the parents as described by Oyedeji [23]. All children who were identified to have high blood pressure, were counselled on lifestyle modification, behavioural changes and referred to specialized clinics for further investigations and follow up.

**Definitions:** Normal blood pressure (NBP) was defined as systolic and diastolic blood pressure that is < 90^th^ percentile for gender, age and height [16]. Elevated blood pressure is when the SBP and/DBP is ≥ 90^th^ percentile to < 95^th^ percentile or 120/80 to 129/ < 80mmHg (whichever was lower) [2]. Stage 1 hypertension is when the SBP and/DBP is ≥ 95^th^ percentile to < 95^th^ percentile + 12 mmHg or 130/80 to 139/89mmHg (whichever was lower) [2]. Stage 2 hypertension is defined as SBP and/DBP ≥ 95^th^ percentile + 12 mmHg or ≥ 140/90 mmHg (whichever was lower) [2]. Normal weight was BMI- for age between the 5^th^ and 85^th^ percentile. Overweight was BMI-for-age between 85^th^ and 95^th^ percentile. Obesity was BMI-for-age above 95^th^ percentile [24].

**Data analysis:** data from the filled questionnaires were entered into a Microsoft Excel spreadsheet, checked, and errors corrected. The dataset was imported into the Statistical Package for Social Science (SPSS) version 23, Armonk, NY: IBM Corp coded, and analyzed. Descriptive analysis was carried out to summarize the socio-demographic and other background variables using mean and standard deviation for quantitative variables and frequency and percentages for categorical variables. Alpha was set at 5%, such that a p-value of less than 0.05 was regarded as statistically significant. The predictors of hypertensive classification were determined using logistic regression analysis. A p-value less than 2 following the univariable regression analysis was included in the multivariate regression model.

**Ethical approval:** ethical approval was obtained from the health research ethics committee of the Lagos University Teaching Hospital with number NHREC/DCST/HERC/2659. Approval with number LG/C530/VI/122 was also obtained from the Lagos State Ministry of Education. Before being enrolled in the study, students and their parents gave written and oral consent/assent.

## Results

### General characteristics of the study population

A total of 1490 students participated, though 1500 were recruited with a response rate of 99.3%. The mean age of the participants was 14.39 ± 2.79 years. There were 744 males and 746 females. The male: female ratio was 1: 1. The highest proportion of participants (649: 43.6%) belonged to the upper socio-economic class. Majority of participants 1245 (83.5%), had normal nutritional status as assessed by Body Mass Index, while 88 (5.9%) were thin. There were 132 (8.9%) overweight and 25 (1.7%) obese participants (Table 1). Based on the 2017 AAP Guideline, three hundred and ninety-eight participants had high blood pressure giving a prevalence rate of 26.7%.

**Table 1:** socio-demographic and BMI Status of participants

Variable	Frequency (n=1490)	Percentage
**Age (years)**		
10-13	590	39.6
14-16	504	33.8
17-19	396	26.6
Mean age ±SD	14.39±2.79	
**Gender**		
Male	744	49.9
Female	746	50.1
Socioeconomic Status		
Upper class (I and II)	649	43.6
Middle class (III)	545	36.6
Lower class (IV and V)	296	19.8
**Body Mass Index (BMI Z score)**		
Thin (<-2SD)	88	5.9
Normal(≥-2SD-≤+1SD)	1245	83
Overweight (> + 1 SD - ≤ + 2 SD) 1	32	8.9
Obese (> + 2 SD)	25	1.7

BMI- Body Mass Index, SD: Standard Deviation

### Pattern of high blood pressure among participants based on the 2017 AAP Guideline

Table 2 shows the pattern of high blood pressure. The prevalence rate of elevated blood pressure was 13.8% while the prevalence rate of stage 1 hypertension was 9.3% and the prevalence rate of stage 2 hypertension was 3.6%. Among the 398 participants that had high blood pressure, slightly more than half (51.5%) had elevated blood pressure. The mean systolic blood pressure was 123.10 ± 3.62 for participants with elevated blood pressure, 132.16 ± 3.27 for participants with stage 1 hypertension and 140.61 ± 8.81 for participants with stage 2 hypertension, while the mean diastolic blood pressure also showed a steady increase and was highest (93.98 ± 4.52) for participants with stage 2 hypertension.

**Table 2:** pattern of high blood pressure among participants based on the 2017 AAP guideline

Variable	Frequency	Percentage of the study population	Percentage of high blood (Mean ±SD)	SBP (Mean ±SD)	DBP (Mean ±SD)
Elevated	205	13.8	51.5	123.10±3.62	68.45±6.83
Stage 1 HTN	139	9.3	34.9	132.16±3.27	80.98±6.46
Stage 2HTN	54	3.6	13.6	140.61±8.81	93.98±4.52
High BP	398	26.7	100	128.64±7.78	76.29±8.19

AAP: American Academy of Pediatrics, SBP: Systolic Blood Pressure, DBP: Diastolic Blood Pressure, HTN: Hypertension, SD: Standard Deviation, CI: Confidence Interval

### Factors associated with hypertension

Logistic regression was applied to identify independent predictors of raised blood pressure. Hypertension was the dependent variable. The independent variables were age, sex and body mass index. Middle and late adolescence, when compared to early adolescence, significantly predicted the likelihood of high blood pressure; aOR 1.78, 95%CI: 1.20 - 2.63, p=0.004 and 3.90 (2.69 - 5.67, p=0.001 respectively]. Similarly, male sex had increased odds for raised blood pressure when compared to females aOR 1.49,95% CI: 1.1 - 2.0, p= 0.009. However, no form of abnormal body mass index (thinness, overweight or obesity) was significantly associated with hypertension (Table 3).

**Table 3:** factors associated with hypertension

Variable	Factors associated with high blood pressure			
	Unadjusted OR (95% CI)	p-value	†Adjusted OR (95% CI)	p-value
**Age group (years)**				
10 – 13®	1		1	
14 – 16	1.63 (1.12 – 2.39)	0.006	1.78 (1.20 – 2.63)	0.004*
17 – 19	3.56 (2.48 – 5.11)	<0.001	3.90 (2.69 – 5.67)	0.001*
**Sex**				
Female ®	1		1	
Male	1.37 (1.03 – 1.83)	0.014	1.49 (1.10 – 2.00)	0.009*
**Body Mass Index**				
Normal ®	1			
Thin	0.78 (0.41 – 1.50)	0.23		
Overweight	1.04 (0.63 – 1.70)	0.44		
Obese	0.48 (0.11 – 2.04)	0.15		

† Adjusted for sex, age and body mass index, ®= reference group. * Significant

## Discussion

The prevalence of high blood pressure, encompassing both elevated blood pressure and hypertension, among adolescents in the current study was found to be 26.7%; associated factors such as abnormal body mass index was observed in 16.5% of participants. The likelihood of being hypertensive was higher in mid-adolescence and late adolescence than in early adolescence; with males more likely to have high BP than females. This 26.7% prevalence is consistent with findings from other countries where the 2017 AAP guideline was used to study high blood pressure among children and adolescents. Prevalence rates of HBP ranging from 24.6% to 25.8% have been reported in studies on children aged 6 to 17 years from China, US, and in an international study involving Poland, Tunisia and some Asian countries [9,10,25,26]. Conversely, the prevalence rate of HBP in the current study is lower than the prevalence rate of 50.4% reported by Abiodun *et al*. [27] among Nigerian adolescents aged 15 to 19 years. This disparity could be due to the participants´ higher age bracket in the latter study. Another plausible explanation is the circumstance the BP was documented. Whereas in the current study the measurements were done in the schools by non-white coat wearing doctors, the Nigerian study by Abiodun *et al*. [27] was retrospective with data documented during a pre-matriculation medical examination for new university students. Anxiety or fear of not being matriculated could have played a role in raising the blood pressure of the students. In this study, the prevalence of elevated blood pressure and hypertension were 13.8% and 12.9% respectively. When compared with the study by Abiodun *et al*. [27] (25.1% and 25.3%), a similar trend of higher prevalence rates was observed. Condren *et al*. [28] in the US also reported a higher prevalence of elevated blood pressure (16.27%) and hypertension (17.85%) in childhood/adolescence using the 2017 guideline.

However, an international study by Yang *et al*.[10], reported a lower rate of 8.6% for elevated blood pressure while Dong *et al*. [26], in China reported a lower rate of 7.9% for hypertension. The smaller sample size and the 10 years minimum age of the current study compared with a sample size of about 50,000 and minimum participant age of 6 years may be responsible for this disparity. That more than a quarter of the adolescents in the present study had high blood pressure is indicative of a high prevalence. This emphasizes the fact that apparently healthy adolescents do have high blood pressure thus underscoring the need for routine blood pressure screening in the school health program. This will enable the identification of adolescents with abnormal values for appropriate intervention, to prevent target organ damage. Another striking finding with respect to age in the present study was that the prevalence of high blood pressure increased progressively with the age group when the 2017 AAP guideline was applied. Further analysis of the observed increase in prevalence rates of high blood pressure upon application of the 2017 guideline in the current study showed that high blood pressure was more likely in males and older adolescents. The findings with respect to males corroborates earlier reports [26-28]. This agrees with the theory that many adolescent boys have a significantly more pronounced rise in blood pressure during puberty [27]. It is pertinent to note that the participants in the current study (10 - 19 years) were in the age of puberty. Sex hormones are thought to play a modifying role in vascular function in hypertension through influencing endothelium-derived contracting factors such as endothelin-1, as well as nitric oxide production.

Higher blood pressure was found to be associated with higher BMI in the present study, as reported in other studies [18,25-27]. Of the 16.5% of participants with abnormal body mass index, 1.7% was obese. The percentage of obese participants in the present study was more than 5 times less than the 9.6% reported by Abiodun *et al*. [27]. Unfortunately, a careful literature search did not reveal other Nigerian studies except a poster presentation by Onagbiye and Toriola [29], which did not provide overall prevalence rates, thus limiting comparisons. In obesity, there is an activation of the sympathetic nervous system, renin-angiotensin-aldosterone system all contributing to the high BP in overweight and obese participants [30]. Obesity is implicated in the development of metabolic syndrome. With the high number of children living with metabolic syndrome globally, highlights the urgent need for multi-sectorial intervention to reduce the global burden of metabolic syndrome and conditions that lead to it, including childhood overweight and obesity [13].

Using logistic regression, age group and male gender are shown to be independent predictors of high blood pressure with the middle and late adolescence age group being classified upwards. This similarity was seen in the study by Condren *et al*. [28], which also found the male gender as independent predictors. The finding in this present study was different from the finding by Sharma *et al*. [31], which showed on logistic regression that the females have at least a 20% higher risk of developing hypertension than males. Logistic regression of the possible predictors of hypertension in the present study revealed that obesity which is a risk factor for hypertension is lower than that seen in the study by Sharma *et al*. [31]. The results of the current study indicate that implementing the 2017 AAP guideline could lead to increased detection of all the categories of high blood pressure. It is hoped that if utilized there will be an increased opportunity for detection of adolescents with high BP and subsequent management to prevent its attendant complications. The implication of which is that the 2017 AAP guideline could be a more sensitive tool for the detection of high blood pressure and fewer children with high blood pressure will be missed. Missing high blood pressure in any child exposes that child to adult hypertension and hypertensive target organ damage including cerebrovascular accident, retinopathy, coronary heart disease myocardial infarction and heart failure, proteinuria and renal failure and atherosclerotic changes [32].

It is imperative that large scale studies are carried out in the community(s) to further explore demographic variables; that may explain the varied prevalence of hypertension and correlates in different parts of Nigeria. The descriptive cross-sectional nature of this study limits its ability to determine causative factors and can only describe associated factors. A multicenter, multi-regional study may have involved children who may provide a different data set for analysis.

## Conclusion

The prevalence rates of high blood pressure, elevated blood pressure and hypertension among adolescents in Mushin LGA using the 2017 AAP Clinical Guideline was high. Implementing the use of the 2017 AAP guideline (a sensitive tool) could lead to increased detection of all the categories of high blood pressure; with an increased opportunity for detection of adolescents with high BP.

### What is known about this topic

- *High blood pressure (HBP), is a growing health problem in adolescents;*
- *To ensure that the adolescent grows into an adult without end organ complications, hypertension needs to detected early and treatment commenced*.

### What this study adds

- *The prevalence of high blood pressure amongst adolescents is high;*
- *Associated factor like age and gender have substantial influence development of hypertension, but this needs to be further investigated considering the increasing incidence of metabolic syndrome and cardiovascular disease*.
